# Assessment of real-time PCR for *Helicobacter pylori* DNA detection in stool with co-infection of intestinal parasites: a comparative study of DNA extraction methods

**DOI:** 10.1186/s12866-020-01824-5

**Published:** 2020-05-24

**Authors:** Martina Leonardi, Giulia La Marca, Barbara Pajola, Francesca Perandin, Marco Ligozzi, Elena Pomari

**Affiliations:** 1 Department of Infectious-Tropical Diseases and Microbiology, IRCCS Sacro Cuore Don Calabria Hospital, Via Don A. Sempreboni, 5 – 37024 Negrar di Valpolicella, Verona, Italy; 2 Department of Diagnostics and Public Health, University of Verona, Verona, Italy

**Keywords:** *Helicobacter pylori*, Intestinal parasites, Co-infection, Stool, Real-time PCR

## Abstract

**Background:**

Many studies reported high prevalence of *H. pylori* infection among patients co-infected with intestinal parasites. Molecular approach for the DNA detection of those microbes in stool have been proposed. However there are a few reports that evaluated the effect of bead-beating in relation to the *H. pylori* outcome. Therefore, we developed and evaluated two TaqMan-based real-time PCR (rt-PCR) qualitative assays for the detection of *ureC* (*glmM*) and *cagA* of *Helicobacter pylori* on DNA extracted by three procedures.

**Results:**

The two PCRs were analysed on 100 stool samples from patients who were screened for intestinal parasites. Three DNA extraction procedures were used: 1) automation with bead beating, 2) automation without bead beating and 3) hand column. The specificity of the new assays was confirmed by sequencing the PCR products and by the lack of cross-reactivity with other bacteria or pathogens DNA. Rt-PCR assays showed a detection limit of 10^4 bacteria/200 mg stool. The *ureC*_PCR with bead beating process was compared to conventional stool antigen test (SAT), with 94.12 and 93.75% of respectively sensitivity and specificity. However, the discordant samples were confirmed by DNA sequencing suggesting a potential higher sensitivity and specificity of PCR.

**Conclusions:**

Our findings showed that the automation with bead-beating –suggested procedure for intestinal parasitic infections- can reach highly sensitive results in *H. pylori* detection on stool compared also with SAT. Thus, this work can provide new insights into the practice of a clinical microbiology laboratory in order to optimize detection of gastro-intestinal infections. Further studies are needed to better define the clinical value of this technique.

## Background

The polymicrobial causes of gastrointestinal disorders have gained tremendous clinical significance [1]. *Helicobacter pylori* and intestinal parasites are common causes of gastrointestinal symptoms and discomfort [2]. In particular, *H. pylori* infection is a major cause of gastric ulcer disease and gastritis in humans and is a risk factor for the development of gastric cancer. It is estimated that *H. pylori* infects more than 50% of the world population with highest burden among developing countries like those in Africa [3]. Intestinal parasites have also a worldwide distribution affecting millions of people globally [4]. Nowadays, the migratory flow has increased also in developed countries. Many studies reported high prevalence of *H. pylori* infection among patients co-infected with intestinal parasites [5–7]. In order to optimize deoxyribonucleic acid (DNA) extraction for the detection of intestinal parasites, previous studies have suggested a supplementary bead-beating step [8–12]. On the other hand, to the best of our knowledge, there are a few publications that evaluated the effect of bead-beating in relation to the *H. pylori* outcome [13–15]. In these studies, the approach of using a stool specimen in a molecular test for non-invasive detection of *H. pylori* DNA has been proposed. However, data on the use of such an approach still require more exploration for its clinical application. Therefore, the aim of this study was to evaluate different methods to improve the detection of *H. pylori* DNA in human stool. We compared the effect of a bead-beating procedure prior to DNA extraction from stool samples with ethanol preservation. We assessed two real-time PCRs (rt-PCR) Taqman for *ureC* (*glmM*) and *cagA,* respectively for the detection of *H. pylori* and for the pathogenicity analysis. For the present study, we collected stool samples from subjects who attended to our hospital earlier and were screened for *H. pylori* by the Stool Antigen Test (SAT) and for intestinal parasites (protozoa and helminths) by multiplex rt-PCRs.

## Results

### Primers and probes optimization for rt-PCR and verification of species-specificity

**Fig. 1:**
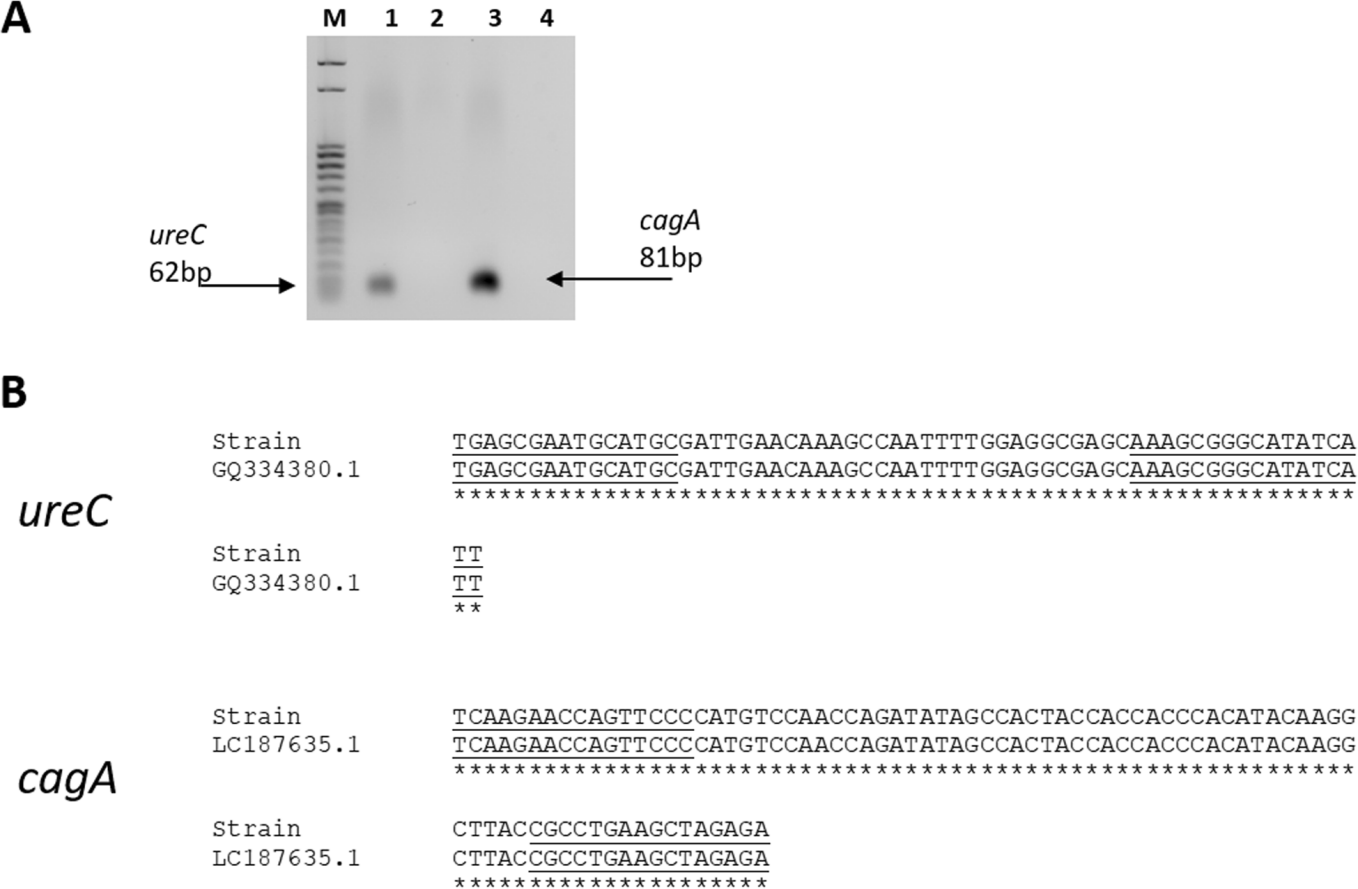
Gel agarose and sequencing results for *H. pylori ureC* and *cagA* PCRs. A) Gel 2% agarose for *ureC* (62 bp) and *cagA* (81 bp). M, DNA marker (50 bp, Sigma); Lane 1, *H. pylori* strain used as positive control for *ureC*_PCR; Lane 2, NTC for *ureC*_PCR; Lane 3, *H. pylori* strain used as positive control for *cagA*_PCR; Lane 4, NTC for *cagA*_PCR. B) Alignment of sequences obtained from *H. pylori* strain (Strain) used as positive control for *ureC* and *cagA* PCRs set-up with 100% identity using BLAST search

We evaluated the optimal amounts of primers/probe by preparing dilution series to determine the minimum concentrations giving the maximum ΔRn (normalized reporter) (supplementary material). All experiments were performed using DNA of a control strain of *H. pylori*. Gel electrophoresis obtained a single band of expected length for the amplicon of *H. pylori* and no signal for the non-template control (Fig. 1a). To determine the species-specificity, the products of conventional PCR for *ureC* and c*agA* were tested by DNA sequencing on the strain used as positive control for the set-up of reactions and for all the analyses. For both target genes *ureC* and *cagA*, the analysis found 100% of identity respectively with *H. pylori* phosphoglucosamine (*glmM*) gene (accession number GenBank: GQ334380.1) and *H. pylori cagA* gene for cytotoxin-associated proteinA (accession number GenBank: LC187635.1) (Fig. 1b). Also, we chose to check the 16S of *H. pylori* as longer fragment (145 base pair (bp)) [16], on *H. pylori* strain and biopsy samples (Fig. 1S). No cross-reaction was seen with bacteria other than *H. pylori*. Thus, using rt-PCR assays, the DNA from strains of *H. pylori* revealed a strong signal with both *ureC*_PCR (mean Ct values = 20.94, standard deviation (SD) = 3.64, *n* = 3) and *cagA*_PCR (mean Ct values = 21.70, SD = 6.25, *n* = 3) and the biopsies revealed a signal of medium intensity with both *ureC*_PCR (mean Ct values = 26.70, SD = 1.20, *n* = 3) and *cagA*_PCR (mean Ct values = 34.61, SD = 5.73, *n* = 3).

### Limit of DNA detection

**Fig. 2:**
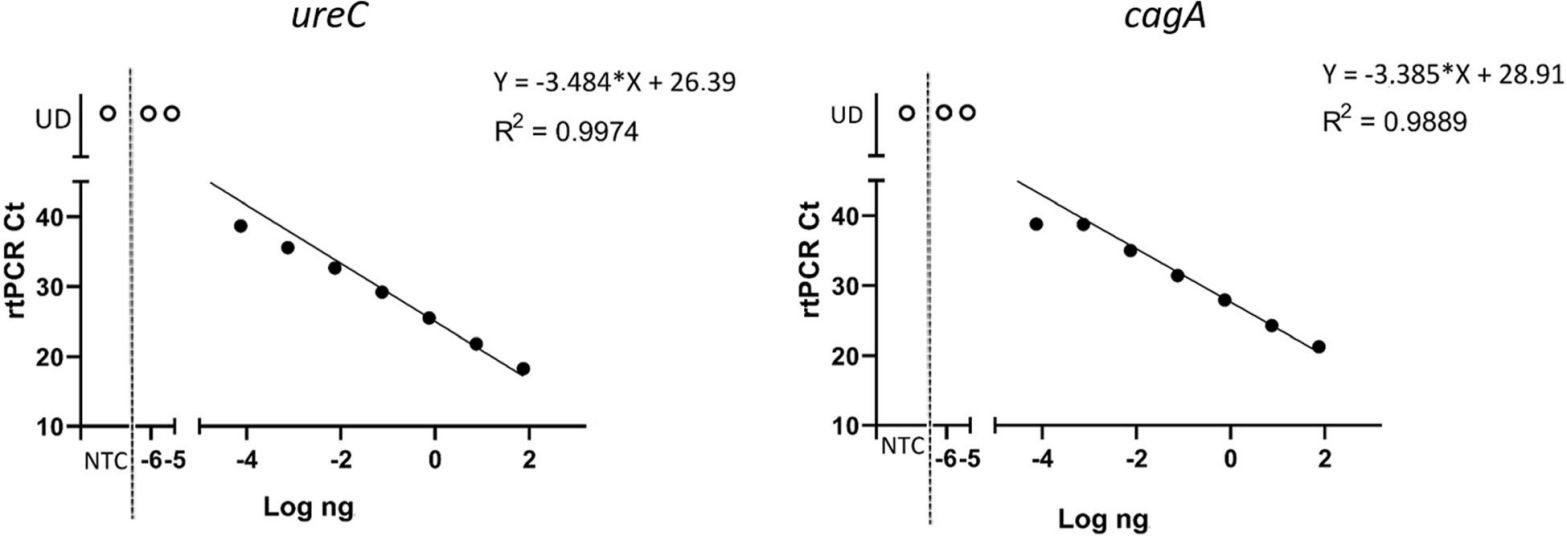
Linear regression analysis for determination of the detection limit with *ureC*_PCR and *cagA*_PCR. UD, undetermined

The results obtained with *ureC*_PCR are reported in the supplementary material. The signal was revealed at a DNA quantity of 0.001875 ng (1.04 × 10^3 copies of DNA) for all 6 replicates. From 6 replicates only one positivity was detected for 0.0001875 ng (1.04 × 10^2 copies of DNA), and at lower concentrations the signal was undetermined (UD), probably due to the low DNA load. In Fig. 2, the linear regression showed good proportionality between the variability of the data with coefficient of determination R^2^ = 0.99. Although the rtPCR was developed to be used as a qualitative and non-quantitative analysis, efficiency was calculated according to the formula Efficiency = − 1 + 10 ^ (− 1 / slope) and a value of 94% was achieved. On the other hand, supplementary material show the results obtained with *cagA*_PCR, and the signal was revealed up to a quantity of 0.01875 ng (1.04 × 10^4 copies of DNA) with a positivity of all 6 replicates and 3 using 0.001875 ng (1.04 × 10^3 copies of DNA). Figure 2 shows the linear regression with an *R*^2^ = 0.98. Also, the efficiency was calculated for the *cagA*_PCR, obtaining a value equal to 97%.

### Limit of detection from stool

Before proceeding with the analysis on the samples of the population included in the study, a verification of the minimum detection limit of the faecal sample was carried out. The supplementary material reports the results. In the case of *ureC* the signal was detected up to a bacteria quantity of 10^4 for all 6 replicates in all three procedures A, B and C. The bacterial quantity of 10^3 was only detected with Procedure B in one replicate from six, probably because of the low DNA load. Similarly, the results for *cagA*_PCR had a signal up to 10^4 bacteria with a positivity of 6/6 replicates in all three procedures.

### Rt-PCR analysis on stool large scale

The *ureC*_PCR and *cagA*_PCR were evaluated on a panel of stool samples positive to *H. pylori* (*n*  =  68) and not (*n*  =  32) as detected previously by SAT. Table 1S reports the mono intestinal parasitic co-infections with *H. pylori*. All the 100 stool samples were analysed by the three different procedures of DNA extraction A, B and C. Since this is a retrospective study, the number of samples extracted with each procedure is not identical. The Ct results are reported in supplementary material. The overall discordant results among the three procedures were *n* = 8 for *ureC* and *n* = 16 for *cagA* (among the SAT positive), and were repeated twice and results were confirmed. The results of the comparison between the reference procedure B (routine procedure in our laboratory) and the others A and C are summarized in Tables 1 and 2. Overall, excluding missing, undetermined and discordant results, we observed not significant difference in Ct mean value by *ureC*_PCR and *cagA*_PCR between procedures. Moreover, we compared SAT results as reference standard for *H. pylori* detection in our study and Procedure B with *ureC*_PCR as routine procedure in our laboratory. We found 94.12% (95% CI 88.53 to 99.71) sensitivity and 93.75% (95% CI 85.36 to 100.00) specificity of *ureC*_PCR. We checked DNA of the discordant samples by 16S PCR and DNA sequencing, and all positive and negative samples were confirmed (Fig. 1S). When available, the DNA was extracted from an additional stool aliquot (apart from sample 56).

**Table 1:** Comparison of mean cycle threshold (Ct) values between DNA extraction procedures for the detection of *H. pylori* on SAT positive (total *n* = 56 samples for *ureC* and *n* = 20 for *cagA*, excluding all missing, undetermined and discordant results)

***PCR*** ***H. pylori***	Ct B vs A	*P* value	Ct B vs C	*P* value	Ct A vs C	*P* value
*ureC*	36.08 vs 36.23	ns	36.08 vs 36.58	ns	36.23 vs 36.58	ns
*cagA*	38.40 vs 38.42	ns	38.40 vs 46.00	ns	39.51 vs 38.42	ns

*ns* not significant by paired Student t-test

**Table 2:** Comparison of mean cycle threshold (Ct) values between DNA extraction procedures for the detection of *H. pylori* on SAT negative. The Ct values were detected on two samples, while the remaining were undetermined. Data are analysed on two positive samples for *ureC* and the only one for *cagA*

***H. pylori***	Ct B vs A	*P* value	Ct B vs C	*P* value	Ct A vs C	*P* value
*ureC*	38.92 vs 35.86	ns	38.92 vs 34.84	ns	35.86 vs 34.84	ns
*cagA*	39.24 vs 45.66	na	39.24 vs 38.18	na	45.66 vs 38.18	na

*ns* not significant by paired Student t-test, *na* not applicable

## Discussion

Molecular methods such as rt-PCR are progressively more used in the clinical microbiology laboratory. In particular, for the intestinal parasitic infections, recent evidences have reported better results by the introduction of a bead-beating step improving the DNA yield [8]. In this context, we wanted to explore also the diagnosis of *H. pylori* on clinical faecal specimens by our routine method of automated DNA extraction that includes bead-beating step, thereby without changing the rt-PCRs outcome targeting other stool parasites. Three procedures (A, B, C) of DNA extraction were evaluated to assess the DNA detection of *H. pylori*. Although the use of bead-beating to isolate DNA of *H. pylori* has been reported in a few publications, details of the benefits are limited and the majority of the studies reported hand column isolation [17–20]. A recent study was performed on 18 faecal specimens collected from five *H. pylori*-infected children and their family members in Japan, and Qiagen column was used after bead-beating [17]. However, in this study, the aim was to investigate the intestinal microbiota of the subject infected with *H. pylori* and the performance of the DNA extraction method was not considered. Additional studies used a bead-beater method to extract total genomic DNA from the gastric biopsy samples by using a combination of the QIAamp DNA isolation kit or automated instruments [18]. To our knowledge, two studies [19, 20] reported details on adding beads in stool for the detection of *H. pylori* DNA by 23S nested PCR. However, column method for DNA isolation was used. In our study, 100 stool samples have been collected from a center in northern Italy known as a reference center for tropical parasitic infections. These stool samples showed great levels of infections with parasites, both helminths and protozoa. The high prevalence of *H. pylori* was confirmed by the detection of it in 68% of the stool samples by SAT. Thus, this population of stool samples was highly suitable for comparing different DNA extraction procedures. Indeed, the majority of prevalence studies of co-infections used serology-based diagnosis for *H. pylori* [21, 22]. Thus, we wanted to implement the *H. pylori* DNA detection on clinical stool as more as suitable technical practice for a molecular parasitology laboratory. In particular, we intended to apply the bead-beating as current and established routine procedure in our department. Indeed, without the addition of bead-beating, the DNA yields of intestinal parasites is generally low [8]. Naturally the yield of *H. pylori* DNA might be expected to be substantially low in human stool specimen [23, 24]. Thus, we combined the demonstrated beneficial effect of ethanol preservation with bead-beating, in particular for helminth infections [25]. We chose to use this preservative solution for all the three procedures of DNA extraction, thus we did not performed a comparison of preservation among them and the internal control did not show inhibition in DNA detection. The *ureC* and *cagA* rt-PCR reactions were designed and assessed in this study. The analyses were conducted on the entire population providing results compared to SAT. First, we observed that *ureC* was detected in 96% of SAT. However, the negative *ureC* samples were confirmed by DNA sequencing, thus indicating that the stool PCR method before *H. pylori* eradication described in this paper appears to be highly sensitive and specific. Also, among the *ureC* positive samples, 54% were also *cagA*+. Of note, *cagA* was detected only in *ureC* positive samples suggesting a good fit between the two molecular assays used in the present study and their agreement on the actual presence of the bacterium DNA. A multiplex assay of *ureC* and *cagA* might be assed in a further work. Thus, our results suggest that both PCRs are suitable tools for detection of *H. pylori* infection on stool before eradication and their optimal time point of application during the follow up of treatment requires further investigation especially for the early eradication [18].

## Conclusions

Overall, our results showed that a bead-beating step prior to automated DNA extraction has relatively minor differences in the output of rt-PCR for *H. pylori* in human stool compared to the automated extraction without bead-beating as well as to the hand column method. This PCR assay appears highly sensitive and specific for the *H. pylori* DNA detection in sample with co-infection of other pathogens. Moreover, a non-invasive molecular assay on clinical stool specimens might be beneficial in detecting not only the infection but also the pathogenicity and the antibiotic resistance of *H. pylori*. However, the cooperation of reference laboratories may be necessary in adding further efforts to optimize the molecular diagnosis of gastro-intestinal infections.

## Methods

### Study design and sample collection

A total of 100 adult migrants and travelers were monitored at IRCCS Sacro Cuore Don Calabria Hospital, Negrar di Valpolicella, Italy, between March 2018 to December 2019. Stool samples were collected from patients retrospectively screened for *H. pylori* by stool antigen test (SAT) and for intestinal parasites by rt-PCR.

### Sample preparation and DNA isolation

Briefly, one aliquot of approximately 1 g was mixed with ethanol solution (96%) for transport at room temperature. Upon arrival at the Department of Infectious-Tropical and Microbiology (DITM), aliquots of approximately 200 mg were prepared and a washing step was applied to the preserved samples to remove the ethanol [25]. Thereafter, the washed samples were suspended in PBS containing 2% polyvinylpolypyrrolidone (pvpp) (Sigma) and then stored at − 20 °C. Three DNA extraction procedures were used (Fig. 2S): Procedure A, DNA extraction performed without bead-beating and using the MagnaPureLC.2 instrument (Roche Diagnostic); Procedure B, DNA extraction with a preliminary step of bead-beating and using the MagnaPureLC.2 instrument; Procedure C, DNA extraction performed by hand using QIAamp Fast DNA Stool Mini Kit (Qiagen). For the bead-beating procedure, 200 mg of stool were transferred into 2-ml screw-capped tube prefilled with ceramic beads (MagNa Lyser Green Beads, Roche), followed by a beating using a homogenization instrument (MagNA Lyser Instrument, Roche). In each sample, Phocin herpes virus-1 (PhHV-1) DNA was included in the isolation lysis buffer, to serve as an internal control [26]. For DNA isolation, in Procedure A and B, 200 μl of stool sample were transferred to the cartridge sample of MagnaPureLC.2 instrument (Roche Diagnostic) following the protocol DNA_I_Blood_Cells_High performance_II, using the DNA isolation kit I (Roche) with a final elution volume of 100 μL. For the Procedure C, 200 μl of stool sample were transferred to the column using QIAamp DNA Stool Mini kit (Qiagen) with a final elution volume of 200 μL, accordingly to the manufacturer’ instructions. All the extracted DNA samples were frozen at − 20 °C until further molecular analysis. As under diagnostic routine conditions according to our protocols, DNA concentrations were not quantified prior to performing the PCR runs.

### Control samples

Positive control *H. pylori* strains (*n* = 3) were kindly provided by prof. Berardino Vaira (Department of Internal Medicine and Gastroenterology, S. Orsola Hospital, Italy), and DNA was extracted by boiling at 95 °C for 10 min. We used also DNA obtained from gastric biopsies (n = 3) positive to *H. pylori* by histology, kindly provided by prof. Giuseppe Zamboni (Department of Anatomic Pathology, IRCCS Sacro Cuore Don Calabria, Italy), and DNA was extracted using QIAamp DNA FFPE Tissue Kit (Qiagen) following the manufacture’ instructions.

### Primers and probes design for *H. pylori*

Two different rt-PCR Taqman assays were used to detect *H. pylori* specific DNA: *ureC* and *cagA*. All primers and probes were selected from gene bank database (Table 3) Nucleotide sequences for several isolates, and only regions with sequence homology of 99% or greater among the various isolates were chosen for primers selection. The PCR primers/probes are designed to amplify a highly conserved 62 bp of *ureC* and 81 bp of *cagA* of the *H. pylori* genome. The primers and probes design was performed using Primer 3 Plus (https://primer3plus.com/cgi-bin/dev/primer3plus.cgi).

**Table 3:** Primers and probes used for *H. pylori* rt-PCR assay

Primer/probe name	Accession number	Primer/probe sequence	Gene target	Amplicon size	Gene position (nt)	Ref
ureC-F	M60398.1	5′-TGAGCGAATGCATGCGATT-3′	*ureC*	62 bp	1447–1466	This study
ureC-R		5′-AATGATATGCCCGCTTTGCT-3’			1489–1509	
FAM-ureC-MGBEQ		5′-ACAAAGCCAATTTTGGAGG-3’			1467–1485	
cagA-F	X70039.1	5′-TCAAGAACCAGTTCCCCATGTC-3’	*cagA*	81 bp	687–709	This study
cagA-R		5′-TCTCTAGCTTCAGGCGGTAAGC-3’			746–768	
HEX-cagA-MGBEQ		5′-ACCAGATATAGCCACTACC-3’			710–730	

### Rt-PCR for *H. pylori*

Both *ureC*_PCR and *cagA*_PCR were performed with a 25 μL reaction mix containing 5 μL DNA, 1 × SsoAdvanced™ Universal Probes Supermix (BioRad), 600 nM of Forward and Reverse primers, 300 nM of probe. The program consisted of an initial step of 2 min at 95 °C followed by 50 cycles of 15 s at 95 °C, 30s at 58 °C and 30s at 72 °C. Amplification, detection and analysis were performed using the CFX96 real-time detection system (Bio-Rad laboratories). No template control (NTC), negative and positive control samples were included in each PCR run. Cycle threshold (Ct) value results were analysed using Bio-Rad CFX software (Manager v3.1). The amplification of individual samples was considered to be hampered by inhibitory factors if the expected Ct-value of 33 in the PhHV-specific PCR [27] was increased by more than 3 cycles [26]. The PhHV PCR showed no significant reduction in Ct value. For each *H. pylori*-specific target, DNA loads were arbitrarily categorized into the following intensity groups: high (Ct < 30), moderate (30⩽Ct⩽35), low (35 < Ct < 50), and negative (≥50 cycles or no amplification detected).

### PCR validation

Primer and probe specificity was checked in silico by BLAST analysis (http://blast.ncbi.nlm.nih.gov/Blast.cgi) and by 2% agarose gel electrophoresis at 100 V for 30 min. The analytical specificity of the PCR was tested on a panel of clinical control samples. The panel included *H. pylori* strain and gastric biopsy samples, all from patients infected with *H. pylori*. To confirm results obtained by the molecular screening for *H. pylori* described above, Sanger sequencing analysis was performed as confirmatory assay. Briefly, DNA target sequences were cloned into *E.coli* (One Shot TOP10) and using GeneArt Seamless Cloning and Assembly kit (Thermofisher). As verification of cloning, HotStarTaq (Qiagen) was used to performed the PCR reactions following the validated conditions. Then, the amplification products, after purification by ExoSap (Applied Biosystems), were sequenced bi-directionally for more accuracy using Big Dye terminator sequencing 3.1 kit (Applied Biosystems) on an ABI Prism 3500 sequencer (Applied Biosystems), following the manufacturer’s instructions. The obtained sequences data were aligned and compared with known sequences data for *H. pylori* (GenBank) using Sequencing Analysis v6 Software (Applied Biosystems) and ClustalW. The specificity of analysis was considered for DNA sequences that align with at least 95% identity to reference sequence.

### Analysis of detection limit

The limit of detection of *ureC*_PCR and *cagA*_PCR was verified using nine serial dilutions (1:10) of DNA with start concentration of 75 ng/μl extracted from the control strain (0.33McFarland corresponding to 10^8/mL cells) of *H. pylori*. The limit of detection was also determined on a 10-fold dilution series of a negative stool sample (negativity was checked for *H. pylori* as well as for all the intestinal parasites considered in the present study) spiked with a quantity of *H. pylori* added (10^6, 10^5, 10^4, 10^3, 10^2 bacteria in 200 mg of stool). DNA was extracted from each dilution using all the three Procedures (A, B and C) (Fig. 2S) and the highest dilution with a positive signal indicated the detection limit. The variation in Ct-values was determined by 6 times within the same run. The coefficient of variation (CV, expressed as %) of the Ct-values was calculated.

### Application of rt-PCR for *H. pylori*

In order to validate the practicality of *H. pylori* DNA detection, we analysed 100 stool samples collected from subjects who attended at our Department. Each stool sample was retrospectively analysed by SAT for *H. pylori* and by rt-PCR examination for intestinal parasites. In particular, according to the routine procedure of our laboratory, molecular diagnostic screening for intestinal parasites was performed by four separate multiplex rt-PCRs for *Entamoeba histolytica*—*Entamoeba dispar*—*Cryptosporidium* spp., for *Giardia intestinalis*—*Dientamoeba fragilis*—*Blastocystis* spp., for *Strongyloides stercoralis*—*Schistosoma* spp—*Hymenolepis nana* and for *Necator americanus*—*Ascaris lumbricoides*—*Ancylostoma duodenale*—*Trichuris trichiura*. Multiplex rt-PCRs were performed adapting the reported protocols [28–36], as summarized in Table 2S. For logistical reasons, the DNA extraction for the molecular analysis of intestinal parasites, was performed by the Procedure B (Fig. 2S) as the routine method used at our laboratory. Thus, for the *H. pylori* DNA detection analysis, we used also two additional and available stored aliquots of each stool sample for the Procedures A and C (Fig. 2S). Since the samples in the study were obtained retrospectively, in one case the stool sample was not available for all three procedures.

### Statistical analysis

All collected data were exported to SAS v9 and GraphPad Prism 8 for statistical analysis and visualization. Descriptive analysis was used to characterise the outcome of each DNA extraction procedure. Student’s t-tests was used for the comparison between procedures of DNA extraction. Negative samples were recoded into an arbitrary value, i.e. Ct 50 for PCR and were excluded in the statistical analysis. A *P*-value < 0.05 was considered to be statistically significant.

## Supplementary information

**Additional file 1: Table S1.** Prevalence of intestinal parasites in subjects positive to *H. pylori* (*n* = 63).

**Additional file 2: Table S2.** Primer/probe sets of four multiplex rt-PCR for intestinal parasites.

**Additional file 3: Figure S1.** Gel agarose and sequencing results for *H. pylori* 16S PCR. A) Gel 2% agarose for 16S PCR (145 bp). M, DNA marker (50 bp, Sigma); Lane 1, first *H. pylori* strain used as positive control for *ureC* and *cagA* PCRs set-up; Lane 2, second *H. pylori* strain; Lane 3, third *H. pylori* strain; Lane 4, first gastric biopsy sample; Lane 5, second gastric biopsy sample; Lane 6, third gastric biopsy sample; Lane 7, stool sample number 72; Lane 8, stool sample number 99; Lane 9; stool sample number 48; Lane 10, stool sample number 56; Lane 11, stool sample number 65; Lane 12, stool sample number 67; Lane 13, NTC. B) Alignment of sequences obtained from *H. pylori* strain used as positive control for *ureC* and *cagA* PCRs set-up (Strain) with 98% identity using BLAST search, from a gastric biopsy (BG) with 97% identity using BLAST search, from stool sample number 72 (72) with 99% identity using BLAST search, from stool sample number 99 (99) with 97% identity using BLAST search.

**Additional file 4: Figure S2.** Flow-chart of the collection and preparations of stool samples. Each preparation procedure is labelled as: Procedure A: DNA extraction was performed on frozen samples without bead-beating and using the MagnaPureLC.2 instrument (Roche Diagnostic); Procedure B: bead-beating was performed before DNA extraction on frozen samples and using the MagnaPureLC.2 instrument (Roche Diagnostic); Procedure C: DNA extraction was performed by hand using QIAamp DNA Stool Mini kit (Qiagen).

**Additional file 5.** Supplementary data set.

## Data Availability

All data generated or analysed during this study are included in this published article (and its supplementary information files).

## Abbreviations

bp: Base pair
Ct: Cycle threshold
DNA: Deoxyribonucleic acid
NTC: No template control
R^2^: Coefficient of determination in linear regression analysis
rt-PCR: Real-time Polymerase chain reaction
SAT: Stool antigen test
SD: Standard deviation
UD: Undetermined

## References

[1] Peters BM, Jabra-Rizk MA, O’May GA, William Costerton J, Shirtliff ME (2012). Polymicrobial interactions: impact on pathogenesis and human disease. Clin Microbiol Rev.

[2] Gravina AG, Zagari RM, De Musis C, Romano L, Loguercio C, Romano M (2018). Helicobacter pylori and extragastric diseases: a review. World J Gastroenterol.

[3] Salih B (2009). Helicobacter pylori infection in developing countries: the burden for how long?. Saudi J Gastroenterology.

[4] Liao CW, Fu CJ, Kao CY, Lee YL, Chen PC, Chuang TW (2016). Prevalence of intestinal parasitic infections among school children in capital areas of the Democratic Republic of São Tomé and Príncipe, West Africa. Afr Health Sci.

[5] Moreira ED, Nassri VB, Santos RS, Matos JF, de Carvalho WA, Silvani CS (2005). Association of Helicobacter pylori infection and giardiasis: results from a study of surrogate markers for fecal exposure among children. World J Gastroenterol.

[6] Ankarklev J, Hestvik E, Lebbad M, Lindh J, Kaddu-Mulindwa DH, Andersson JO (2012). Common Coinfections of Giardia intestinalis and helicobacter pylori in non-symptomatic Ugandan children. PLoS Negl Trop Dis.

[7] Yakoob J, Abbas Z, Khan R, Tariq K, Awan S, Beg MA (2018). Association of Helicobacter pylori and protozoal parasites in patients with chronic diarrhoea. Br J Biomed Sci.

[8] Kaisar MMM, Brienen EAT, Djuardi Y, Sartono E, Yazdanbakhsh M, Verweij JJ (2017). Improved diagnosis of Trichuris trichiura by using a bead-beating procedure on ethanol preserved stool samples prior to DNA isolation and the performance of multiplex real-time PCR for intestinal parasites. Parasitology..

[9] Ayana M, Cools P, Mekonnen Z, Biruksew A, Dana D, Rashwan N (2019). Comparison of four DNA extraction and three preservation protocols for the molecular detection and quantification of soil-transmitted helminths in stool. PLoS Negl Trop Dis.

[10] Andersen LOB, Röser D, Nejsum P, Nielsen HV, Stensvold CR (2013). Is supplementary bead beating for DNA extraction from nematode eggs by use of the nuclisens easymag protocol necessary. J Clin Microbiol.

[11] Liu J, Gratz J, Amour C, Nshama R, Walongo T, Maro A (2016). Optimization of quantitative PCR methods for enteropathogen detection. PLoS One.

[12] Platts-Mills JA, Gratz J, Mduma E, Svensen E, Amour C, Liu J (2014). Association between stool enteropathogen quantity and disease in Tanzanian children using TaqMan Array cards: a nested case-control study. Am J Trop Med Hyg..

[13] Beckman E, Saracino I, Fiorini G, Clark C, Slepnev V, Patel D (2017). A novel stool PCR test for helicobacter pylori may predict clarithromycin resistance and eradication of infection at a high rate. J Clin Microbiol.

[14] Beer-Davidson G, Hindiyeh M, Muhsen K (2018). Detection of Helicobacter pylori in stool samples of young children using real-time polymerase chain reaction. Helicobacter.

[15] Scaletsky ICA, Aranda KRS, Garcia GT, Gonçalves MEP, Cardoso SR, Iriya K (2011). Application of real-time PCR stool assay for helicobacter pylori detection and clarithromycin susceptibility testing in Brazilian children. Helicobacter..

[16] Rasmussen LT, de Labio RW, Neto AC, Silva LC, Queiroz VF, Smith MAC (2012). Detection of helicobacter pylori in gastric biopsies, saliva and dental plaques of dyspeptic patients from Marília, São Paulo, Brazil: presence of vacA and cagA genes. J Venom Anim Toxins Incl Trop Dis.

[17] Osaki T, Zaman C, Yonezawa H, Lin Y, Okuda M, Nozaki E (2018). Influence of intestinal indigenous microbiota on intrafamilial infection by helicobacter pylori in Japan. Front Immunol.

[18] Puz S, Innerhofer A, Ramharter M, Haefner M, Hirschl AM, Kovách Z (2008). A novel noninvasive genotyping method of helicobacter pylori using stool specimens. Gastroenterology..

[19] Noguchi N, Rimbara E, Kato A, Tanaka A, Tokunaga K, Kawai T (2007). Detection of mixed clarithromycin-resistant and -susceptible helicobacter pylori using nested PCR and direct sequencing of DNA extracted from faeces. J Med Microbiol.

[20] Rimbara E, Sasatsu M, Graham DY (2013). PCR detection of helicobacter pylori in clinical samples. Methods Mol Biol.

[21] Seid A, Tamir Z, Kasanew B, Senbetay M (2018). Co-infection of intestinal parasites and helicobacter pylori among upper gastrointestinal symptomatic adult patients attending Mekanesalem hospital, Northeast Ethiopia. BMC Res Notes.

[22] Ek C, Whary MT, Ihrig M, Bravo LE, Correa P, Fox JG (2012). Serologic evidence that Ascaris and toxoplasma infections impact inflammatory responses to helicobacter pylori in Colombians. Helicobacter..

[23] Dolan B, Burkitt-Gray L, Shovelin S, Bourke B, Drumm B, Rowland M (2018). The use of stool specimens reveals helicobacter pylori strain diversity in a cohort of adolescents and their family members in a developed country. Int J Med Microbiol.

[24] Monteiro L, Gras N, Vidal R, Cabrita J, Mégraud F (2001). Detection of helicobacter pylori DNA in human feces by PCR: DNA stability and removal of inhibitors. J Microbiol Methods.

[25] ten Hove RJ, Verweij JJ, Vereecken K, Polman K, Dieye L, van Lieshout L (2008). Multiplex real-time PCR for the detection and quantification of Schistosoma mansoni and S. haematobium infection in stool samples collected in northern Senegal. Trans R Soc Trop Med Hyg.

[26] Niesters HGM (2002). Clinical virology in real time. J Clin Virol.

[27] Perandin F, Pomari E, Bonizzi C, Mistretta M, Formenti F, Bisoffi Z (2018). Assessment of real-time polymerase chain reaction for the detection of trichostrongylus spp. DNA from human fecal samples. Am J Trop Med Hyg.

[28] Verweij JJ, Schinkel J, Laeijendecker D, Van Rooyen MAA, Van Lieshout L, Polderman AM (2003). Real-time PCR for the detection of Giardia lamblia. Mol Cell Probes.

[29] Verweij JJ, Oostvogel F, Brienen EAT, Nang-Beifubah A, Ziem J, Polderman AM (2003). Short communication: prevalence of Entamoeba histolytica and Entamoeba dispar in northern Ghana. Trop Med Int Heal.

[30] Verweij JJ, Mulder B, Poell B, van Middelkoop D, Brienen EAT, van Lieshout L (2007). Real-time PCR for the detection of Dientamoeba fragilis in fecal samples. Mol Cell Probes.

[31] Verweij JJ, Canales M, Polman K, Ziem J, Brienen EAT, Polderman AM (2009). Molecular diagnosis of Strongyloides stercoralis in faecal samples using real-time PCR. Trans R Soc Trop Med Hyg.

[32] Jothikumar N, Da Silva AJ, Moura I, Qvarnstrom Y, Hill VR (2008). Detection and differentiation of Cryptosporidium hominis and Cryptosporidium parvum by dual TaqMan assays. J Med Microbiol.

[33] Obeng BB, Aryeetey YA, De Dood CJ, Amoah AS, Larbi IA, Deelder AM (2008). Application of a circulating-cathodic-antigen (CCA) strip test and real-time PCR, in comparison with microscopy, for the detection of Schistosoma haematobium in urine samples from Ghana. Ann Trop Med Parasitol.

[34] Stensvold CR, Ahmed UN, Andersen LOB, Nielsen HV (2012). Development and evaluation of a genus-specific, probe-based, internal-process-controlled real-time PCR assay for sensitive and specific detection of Blastocystis spp. J Clin Microbiol.

[35] Liu J, Gratz J, Amour C, Kibiki G, Becker S, Janaki L (2013). A laboratory-developed taqman array card for simultaneous detection of 19 enteropathogens. J Clin Microbiol.

[36] Llewellyn S, Inpankaew T, Nery SV, Gray DJ, Verweij JJ, Clements ACA (2016). Application of a multiplex quantitative PCR to assess prevalence and intensity of intestinal parasite infections in a controlled clinical trial. PLoS Negl Trop Dis.

