# Supplementary material for: Assessment of real-time PCR for Helicobacter pylori DNA detection in stool with co-infection of intestinal parasites: a comparative study of DNA extraction methods
Source: BMC Microbiol. 2020 May 24;20:131. doi: 10.1186/s12866-020-01824-5 (PMC7247253; doi:10.1186/s12866-020-01824-5)
Supplement: Supplementary file 1 — Additional file 1: Table S1. Prevalence of intestinal parasites in subjects positive to H. pylori (n = 63). [file 12866_2020_1824_MOESM1_ESM.docx]

Table 1S. Prevalence of intestinal parasites in subjects positive to *H. pylori* (n=63).

| **Mono co-infection with *H. pylori*** | **Total**  **n** |
| --- | --- |
| *Blastocystis* | 36 |
| *E. dispar* | 3 |
| *D. fragilis* | 6 |
| *G. intestinalis* | 3 |
| *H. nana* | 1 |
| *N. americanus* | 3 |
| *S. mansoni* | 9 |
| *S. stercoralis* | 2 |
