# Supplementary material for: Assessment of real-time PCR for Helicobacter pylori DNA detection in stool with co-infection of intestinal parasites: a comparative study of DNA extraction methods
Source: BMC Microbiol. 2020 May 24;20:131. doi: 10.1186/s12866-020-01824-5 (PMC7247253; doi:10.1186/s12866-020-01824-5)
Supplement: Supplementary file 2 — Additional file 2: Table S2. Primer/probe sets of four multiplex rt-PCR for intestinal parasites. [file 12866_2020_1824_MOESM2_ESM.docx]

**Table 2S.** Primer/probe sets of four multiplex rt-PCR for intestinal parasites

| **Multiplex** | **Parasite** | **Primers/probe** | **Ref** |
| --- | --- | --- | --- |
| 1 | *Entamoeba histolytica* | For 5’-ATTGTCGTGGCATCCTAACTCA-3’  Rev 5’-GCGGACGGCTCATTATAACA-3’  Probe 5’-FAM-TCATTGAATGAATTGGCCATTT-3’-MGB | [29] |
|  | *Entamoeba dispar* | For 5’-ATTGTCGTGGCATCCTAACTCA-3’  Rev 5’-GCGGACGGCTCATTATAACA-3’  Probe 5’-HEX-TTACTTACATAAATTGGCCACTTTG-3’-MGB | [29] |
|  | *Cryptosporidium spp.* | For 5’-ATGAGCGGGTAACGGGGAAT-3’  Rev 5’-CCAATTACAAAACCAAAAAGTCC-3’  Probe 5’-CY55-CGCGCCTGCTGCCTTCTTTAGATG-3’-BBQ | [32] |
| 2 | *Giardia intestinalis* | For 5’-GACGGCTCAGGACAACGGTT-3’  Rev 5’-TTGCCAGCGGTGTCCG-3’  Probe 5’-CY55-CCCGCGGCGGTCCCTGCTAG-3’-BHQ | [28] |
|  | *Dientamoeba fragilis* | For 5’-CAACGGATGTCTTGGCTCTTTA-3’  Rev 5’-TTGCCAGCGGTGTCCG-3’  Probe 5’-HEX-CAATTCTAGCCGCTTAT-3’-MGB | [30] |
|  | *Blastocystis spp.* | For 5’-GGTCCGGTGAACACTTTGGATTT-3’  Rev 5’-CCTACGGAAACCTTGTTACGACTTCA-3’  Probe 5’-FAM-TCGTGTAAATCTTACCATTTAGAGGA-3’-MGB | [34] |
| 3 | *Strongyloides stercoralis* | For 5’-GAATTCCAAGTAAACGTAAGTCATTAGC-3’  Rev 5’-TGCCTCTGGATATTGCTCAGTTC-3’  Probe 5’-FAM-ACACACCGGCCGTCGCTGC-3’-BHQ | [31] |
|  | *Schistosoma spp* | For 5’-GGTCTAGATGACTTGATYGAGATGCT-3’  Rev 5’-TCCCGAGCGYGTATAATGTCATTA-3’  Probe 5’-FAM-TGGGTTGTGCTCGAGTCGTGGC-3’-BHQ | [33] |
|  | *Hymenolepis nana* | For (Hna-F1) 5’-CATTGTGTACCAAATTGATGATGAGTA-3’  Rev (Hna-R1) 5’-CAACTGACAGCATGTTTCGATATG-3’  Probe (Hna-1-probe)  5’-YAKYE-CGTGTGCGCCTCTGGCTTACCG-3’-BHQ | Developed by Dr Verweij JJ |
| 4 | *Necator americanus* | For 5’-CTGTTTGTCGAACGGTACTTGC-3’  Rev 5’-ATAACAGCGTGCACATGTTGC-3’  Probe 5’-FAM-CTGTACTACGCATTGTATAC-3’-MGB | [36] |
|  | *Ascaris lumbricoides* | For 5’-GTAATAGCAGTCGGCCGGTTTCTT-3’  Rev 5’-GCCCAACATGCCACCTATTC-3’  Probe 5’-ROX-TTGGCGGACAATTGCATGCGAT-3’-BHQ | [36] |
|  | *Ancylostoma duodenale* | For 5’-GAATGACAGCAAACTCGTTGTTG-3’  Rev 5’-ATACTAGCCACTGCCGAAACGT-3’  Probe 5’-HEX-ATCGTTTACCGACTTTAG-3’-MGB | [36] |
|  | *Trichuris trichiura* | For 5’-TCCGAACGGCGGATCA-3’  Rev 5’-CTCGAGTGTCACGTCGTCCTT-3’  Probe 5’-CY55-CGATGGTACGCTACGTGCTTACCATGG-3’-BHQ | [35] |
