# Supplementary figures and images for: Assessment of real-time PCR for Helicobacter pylori DNA detection in stool with co-infection of intestinal parasites: a comparative study of DNA extraction methods
Source: BMC Microbiol. 2020 May 24;20:131. doi: 10.1186/s12866-020-01824-5 (PMC7247253; doi:10.1186/s12866-020-01824-5)

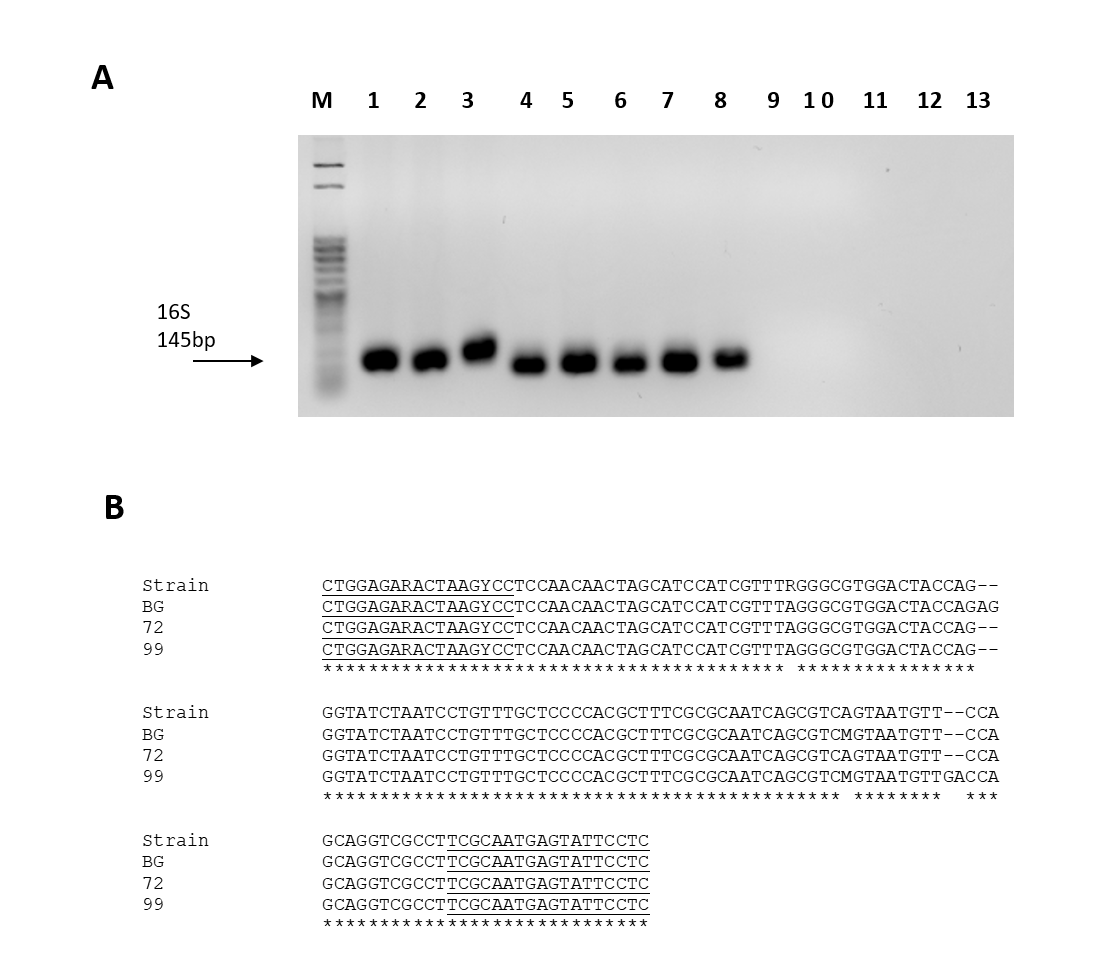

Supplement: Supplementary file 3 — Additional file 3: Figure S1. Gel agarose and sequencing results for H. pylori 16S PCR. A) Gel 2% agarose for 16S PCR (145 bp). M, DNA marker (50 bp, Sigma); Lane 1, first H. pylori strain used as positive control for ureC and cagA PCRs set-up; Lane 2, second H. pylori strain; Lane 3, third H. pylori strain; Lane 4, first gastric biopsy sample; Lane 5, second gastric biopsy sample; Lane 6, third gastric biopsy sample; Lane 7, stool sample number 72; Lane 8, stool sample number 99; Lane 9; stool sample number 48; Lane 10, stool sample number 56; Lane 11, stool sample number 65; Lane 12, stool sample number 67; Lane 13, NTC. B) Alignment of sequences obtained from H. pylori strain used as positive control for ureC and cagA PCRs set-up (Strain) with 98% identity using BLAST search, from a gastric biopsy (BG) with 97% identity using BLAST search, from stool sample number 72 (72) with 99% identity using BLAST search, from stool sample number 99 (99) with 97% identity using BLAST search. [file 12866_2020_1824_MOESM3_ESM.tif]

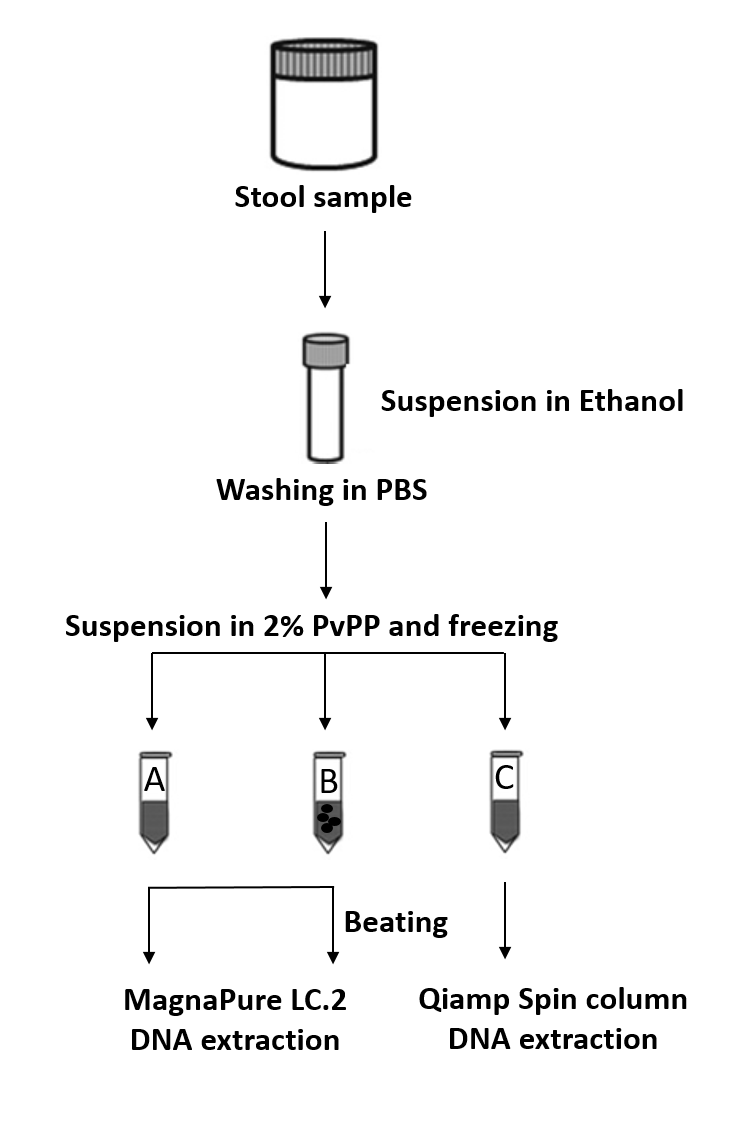

Supplement: Supplementary file 4 — Additional file 4: Figure S2. Flow-chart of the collection and preparations of stool samples. Each preparation procedure is labelled as: Procedure A: DNA extraction was performed on frozen samples without bead-beating and using the MagnaPureLC.2 instrument (Roche Diagnostic); Procedure B: bead-beating was performed before DNA extraction on frozen samples and using the MagnaPureLC.2 instrument (Roche Diagnostic); Procedure C: DNA extraction was performed by hand using QIAamp DNA Stool Mini kit (Qiagen). [file 12866_2020_1824_MOESM4_ESM.tif]
